# Large Language Model–Assisted Thematic Coding in Medical Education Research: Comparative Methodological Study

**DOI:** 10.2196/85572

**Published:** 2026-08-25

**Authors:** Alexa DeRegnaucourt, Katherine Miller Jennings, Andrew Zahn, Neal Taliwal, Matthew Kelleher, Christine Yang Zhou, Seth Overla, Sally A Santen, Danielle Elliott Weber, Laurah Turner

**Affiliations:** 1 College of Medicine, University of Cincinnati, Cincinnati, OH, United States; 2 Department of Pediatrics, College of Medicine, University of Cincinnati, Cincinnati, OH, United States; 3 Department of Internal Medicine, College of Medicine, University of Cincinnati, Cincinnati, OH, United States; 4 Division of Pulmonary and Critical Care, College of Medicine, University of Cincinnati, Cincinnati, OH, United States; 5 Department of Medical Education, College of Medicine, University of Cincinnati, Cincinnati, OH, United States; 6 Department of Emergency Medicine, College of Medicine, University of Cincinnati, Cincinnati, OH, United States; 7 Department of Biostatistics, Health Informatics and Data Science, College of Medicine, University of Cincinnati, Medical Sciences Building G453D, Cincinnati, OH, United States, 1 513-330-3999

**Keywords:** AI, large language model, generative AI, qualitative, learning tool

## Abstract

**Background:**

While large language model (LLM)–assisted qualitative analysis could improve the efficiency and scalability of feedback-driven curricular refinement in medical education, how best to leverage LLMs for qualitative analysis while ensuring quality outputs remains an open question. Prior work has demonstrated the feasibility of using LLMs for inductive and deductive coding tasks, but more needs to be known about how LLM-assisted thematic coding can best be deployed in a medical education context to maximize its strengths and guard against its weaknesses.

**Objective:**

Our study evaluated LLM performance in inductive code generation and in the deductive application of a human codebook, using a student focus-group transcript, to propose a model for AI collaboration in qualitative analysis.

**Methods:**

The qualitative data for this study consisted of a 1-hour focus group with 4 second-year medical students discussing a required AI-driven clinical-scenario tool (2-Sigma). Three human coders conducted an inductive thematic analysis. Using the same transcript, GPT-4o (version gpt-4o-2024-11-20; OpenAI) generated inductive codes and applied the human codebook deductively. The researchers compared the alignment between the AI inductive codes and the human consensus codebook using 3 categories: agreement, reasonable alternative, and not reasonable. Interrater reliability of AI deductive coding was evaluated using percent agreement and Cohen κ, with textual audits of discrepancies, including “misses” (failed to apply appropriate codes) and “misfires” (inappropriately applied codes). Analysis took place between February and July 2025.

**Results:**

In the inductive condition, GPT-4o generated 137 initial codes, of which 31.4% (n=43) demonstrated agreement with human codes, 26.3% (n=36) represented reasonable alternatives, and 42.3% (n=58) were classified as not reasonable. In the deductive condition, mean percent agreement for AI application of human codes was 96% (SD 4%, range 79%‐100%) and the mean κ was 0.71 (SD 0.26, range 0‐1.00). Of all 2352 coding decisions, there were 57 (2.4%) misfires and 28 (1.2%) misses; common patterns included overinterpretation of tone, failure to recognize continued ideas across excerpts, and difficulty distinguishing hypothetical vs experienced features. Based on our findings, we suggest a roadmap that retains human interpretive control while leveraging AI scalability: humans first develop a contextually grounded codebook through inductive analysis, then use AI both as a creative partner to surface alternative codes and as a tool to apply the validated codebook across the dataset.

**Conclusions:**

With targeted human oversight, an LLM reliably applied an existing codebook and generated additional inductive codes. These findings support a proposed workflow in which AI serves as an additional perspective within human-driven qualitative analysis, offering a scalable adjunct for qualitative analysis in medical education. Validation across larger and more diverse datasets will help confirm the generalizability of this approach.

## Introduction

The question of whether and how to use AI for qualitative analysis in medical education is as promising as it is fraught. On the one hand, large language model (LLM)–assisted analysis offers the potential to improve the efficiency and scalability of feedback-driven curricular refinement, which is often constrained by the time-intensive and resource-intensive nature of traditional qualitative methods. On the other hand, the use of LLMs in qualitative research introduces important methodological and epistemological challenges.

Although current research emphasizes “keeping the human in the loop” to ensure quality outputs and preserve the interpretivist and constructivist bones of qualitative research, how to do so remains an open question. Much of the literature has reported interrater agreement between AI and humans on deductive coding tasks, with a need for in-depth error analysis of AI coding decisions [1,2]. In the inductive realm, prior studies comparing human and AI-generated themes have demonstrated overlap [3-5], with the latter hindered by contextual limitations [4,6], a lack of interpretive richness [4], and critical omissions [5]. AI has clearly demonstrated itself to be capable of generating codes [5]; however, there is a need to understand how AI-generated codes could be consolidated with human codes.

Although many studies have compared AI and human performance in qualitative analysis, few have made this comparison through the lens of medical education [7,8]. Evaluating AI performance in this environment is especially important, since there are unique language, cultural norms, and conceptual frameworks. Meaningful qualitative research in medical education is highly contextual and socially situated, and therefore domain-specific evaluation is necessary to determine how AI can or should assist in the process. As medical schools iteratively use student feedback in curricular decisions, the potential to implement AI for efficient, less resource-intensive analysis of this feedback is growing. However, there is a gap in knowledge regarding the balance of human vs AI involvement in qualitative coding, especially in the context of medical education. A better understanding of the specific capabilities and limitations of AI coding of qualitative data can help educators strike an optimal balance in the responsible and critical use of AI.

In this study, we evaluated GPT-4o’s (version gpt-4o-2024-11-20; OpenAI) capabilities in both inductive code generation and deductive application of a single human-generated codebook. This human-generated codebook was created through independent coding of a focus-group transcript by multiple human coders, condensed into a single human “consensus codebook” through discussion and reflexive comparison among the coders.

The inductive coding capabilities of GPT-4o were assessed by comparing its independently generated codes to the human-generated codes using an in-depth textual review of its coding decisions by 2 human raters and categorizing these by level of agreement. The deductive coding capabilities of GPT-4o were assessed by prompting it to apply the human consensus codebook to the transcript and then using 2 traditional methods of interrater reliability, Cohen κ and simple percent agreement, to quantify agreement. These comparisons were used to better understand the strengths and weaknesses of AI and to propose a model for collaboration between AI and humans in the coding of qualitative data that optimizes effectiveness while ensuring responsible use. Importantly, this is a methods study; our goal was not to conduct a full qualitative research project but rather to examine whether AI can augment the coding process, a critical but resource-intensive step in qualitative research.

## Methods

### Source Data

An AI-driven clinical scenario learning tool (2-Sigma) [9] was implemented within the required preclinical curriculum at the University of Cincinnati College of Medicine in August 2023. Within 1 month of implementation, a voluntary and uncompensated 1-hour focus group was hosted with 4 second-year medical students. The focus group facilitator used a semistructured interview guide to explore student experiences with the tool.

Audio recordings were transcribed using the ElevenLabs Scribe automatic speech recognition model. We deidentified the data, resolved overlapping speech segments, and removed facilitator contributions to focus the analysis on student perspectives. The final transcript was organized into “excerpts.” An excerpt is defined herein as an uninterrupted student response within the focus group transcript. In other words, “excerpt” is used to describe 1 participant’s turn speaking. The transcript contained 6488 words and 48 excerpts (mean 135 words, SD 99.3; range 2‐400). We selected this unit of analysis because our objective was to compare whether human and AI coders identified the presence of codes within naturally occurring student contributions during focus group discussion, rather than perform sentence-level discourse analysis. This approach preserved the conversational context of each student response while allowing coders to identify multiple concepts within a single contribution. Accordingly, one excerpt may receive multiple codes if it reflects more than one concept.

### Human Thematic Analysis

We conducted an inductive thematic analysis informed by the 6-phase framework of Braun and Clarke [10], using an iterative, consensus-oriented codebook approach. Three coders (KMJ, AD, and NT) independently and inductively coded the transcripts at the level of individual excerpts. The coders then met to develop a shared codebook through discussion and reflexive comparison of interpretations. Differences in coding decisions were discussed with attention to the unique experiences of each coder, such as stage of medical training, experience with AI, and personal experience with the 2-Sigma tool. These coding differences were addressed via group reconciliation to (1) settle on one of the existing codes, (2) create a unique code to encompass similar ideas, or (3) keep the 2 differing codes to expand interpretive richness.

Codes were refined iteratively to improve clarity, conceptual coherence, mutual exclusivity, and completeness. To minimize conceptual overlap between codes, coders iteratively reviewed the codebook and compared excerpts assigned to similar codes. Through this consensus-based discussion, code boundaries were redefined, operational definitions revised, and inclusion and exclusion criteria clarified to improve conceptual distinctness and consistency. When substantial redundancy was identified, codes were merged or narrowed to ensure that each represented a discrete analysis concept while preserving relevant nuance.

After group reconciliation, 49 codes were agreed upon to represent the transcript. The group then created operational definitions with inclusion and exclusion criteria for each code and added example quotes from the transcript. Two coders (KMJ and AD) then applied this final codebook to the entire transcript and, again, met to discuss their assignment of codes and come to agreement. The human codebook is available in Multimedia Appendix 1.

All 3 coders (KMJ, AD, and NT) were novices. As medical students with experience using the 2-Sigma learning tool, the coders were well positioned to interpret student feedback. The coders did not participate in the focus groups.

### AI Model Selection and Technical Implementation

We used GPT-4o for inductive and deductive coding between February 2025 and July 2025. GPT-4o was chosen due to its availability as a relevant and free platform, released less than 1 year prior to the study start. Prompts were developed iteratively to address anticipated and unanticipated AI limitations. These implementation challenges and the strategies used to address them are discussed further in the *Discussion* section.

Analysis was conducted using custom Python scripts with automated logging of API calls, responses, and processing times. An API call is a request sent from one program to another; in this case, a request sent from Python to GPT-4o. No memory is retained between prompts except for what is explicitly included in the prompt.

### AI Inductive Code Generation

**Figure 1.**
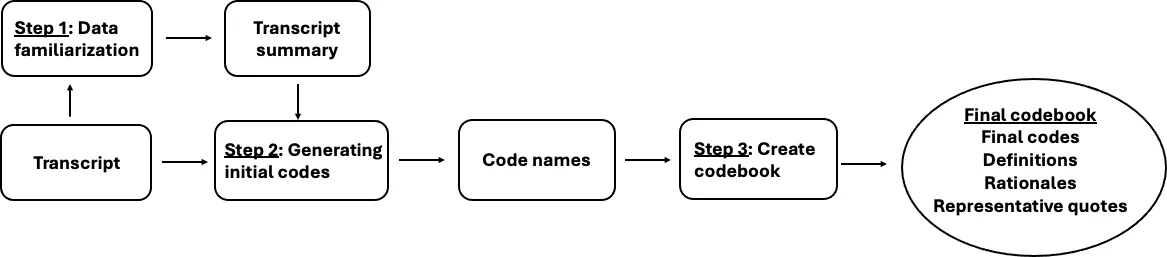
Inductive AI coding structure. This figure demonstrates the key steps of AI inductive code generation from a focus group transcript, including familiarization with the data, generating initial codes, and final codebook generation including definitions, rationales, and representative quotes.

For inductive coding, we designed a 3-step process that mirrored human thematic analysis phases (Figure 1).

#### Step 1: Data Familiarization

GPT-4o generated a preliminary summary of the entire transcript, identifying broad themes, patterns, and key concepts [5]. We prompted the model with a 386-token prompt applied to the full dataset of 18,263 tokens, generating a 525-token summary. This step documented the model’s initial interpretation of the full dataset before coding.

#### Step 2: Generating Initial Codes

The transcript was organized into 48 excerpts after overlapping speech was resolved and facilitator contributions were removed. GPT-4o was prompted to generate 1 to 3 codes per excerpt to ensure systematic attention to all contents [5]. We used an 845-token prompt and systematically processed each of the 48 excerpts, with individual iterations averaging 1024 tokens. The decision to guide the AI with 1 to 3 codes per excerpt was chosen to provide sufficient coverage based on our experience coding the transcript. For each code, GPT-4o also provided a definition and a brief rationale for each code to increase transparency.

#### Step 3: Codebook Generation

We prompted GPT-4o to incorporate its codes from step 2 into a final codebook with a code name, a definition, a rationale, and a representative quote from the transcript. This required approximately 12,000 tokens per code.

The full prompt for the inductive condition is available in Multimedia Appendix 2 via the GitHub link.

### Evaluation of AI Inductive Coding

All GPT-4o inductively generated codes were compared to the human codes by 2 human raters (KMJ and AD). This comparison was done by categorizing each AI code into 3 levels of agreement with human codes:

- “Agreement”: aligned with a human code
- “Reasonable alternative”: textually supported concepts distinct from those in the human codebook, including granular or tangential but accurate ideas
- “Not reasonable”: lacking textual support or containing logical inconsistencies

The categorization of each code was agreed upon by the 2 human raters (KMJ and AD) to create the final comparisons of human vs AI inductive coding. An example code and rationale for each category of alignment are provided in Multimedia Appendix 3.

### AI Deductive Code Generation

**Figure 2.**
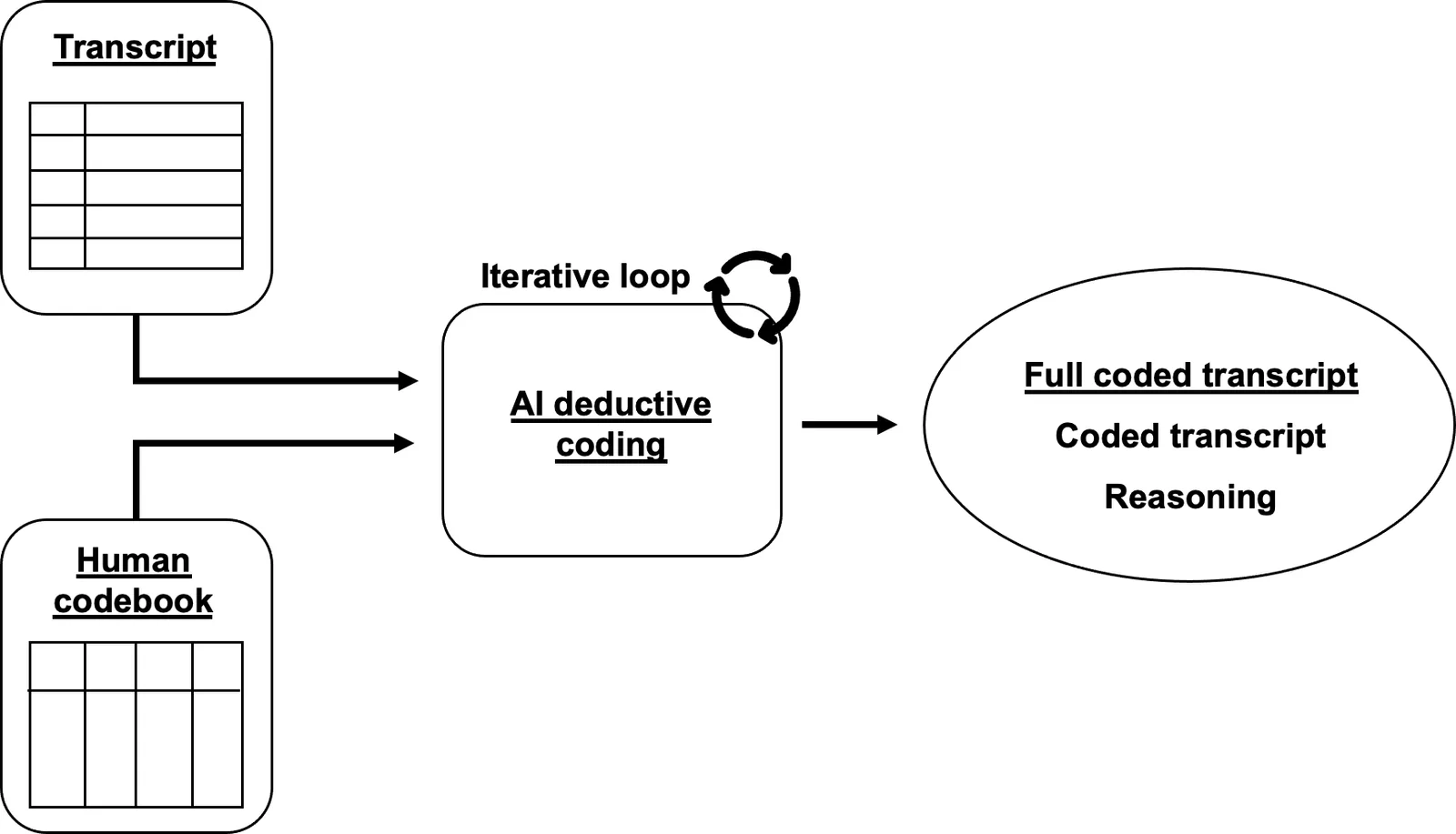
Deductive AI coding structure and prompt. This figure demonstrates the key steps of AI deductive application of a human codebook to a student focus-group transcript. The AI was asked to make a coding decision for each individual code-excerpt pair. If a code was applied, the AI provided reasoning for why it applied the code.

For the deductive condition, GPT-4o was provided with the human codebook and prompted to apply codes to the transcript (Figure 2). To ensure that the AI considered every excerpt-code combination and did not ignore parts of the transcript or codebook, we prompted the AI to analyze each excerpt against each code individually. For each excerpt-code combination, the AI was instructed to decide if the code was applied and provide reasoning if the code was applied. This also solved the limitation of token limits, prohibiting the full transcript and codebook from being provided at once. The full deductive condition prompt is available in Multimedia Appendix 4 at the GitHub link.

### Evaluation of AI Deductive Coding

We assessed AI’s fidelity in applying the human codebook using 2 complementary interrater reliability measures: Cohen κ and simple percent agreement. Both measures of interrater reliability were calculated for each of the 49 human codes to quantify the level of agreement between the human and the AI in applying that code to the transcript. Applying 2 indices, rather than just 1, is the preferred approach, given that each has weaknesses [11].

Percent agreement represents the sum of coding agreements over the total number of items [11,12]. A minimum of 80% is the established benchmark for percent agreement [11,12]. For Cohen κ, we adopted the interpretation offered by Landis and Koch [13] and selected the established benchmark of >0.60, corresponding to “substantial” agreement.

For codes failing to meet reliability benchmarks, one rater (KMJ) reviewed coding discrepancies to identify specific patterns in AI performance, categorizing errors as “misses” (failed to apply appropriate codes) or “misfires” (inappropriately applied codes).

### Statistical Analysis

For interrater reliability calculations, we used the scikit-learn library to compute Cohen κ and developed custom scripts to calculate percent agreement. All statistical analyses included CIs and descriptive statistics.

### Timeline and Resource Tracking

Human coders retroactively estimated the time commitment for each part of the manual qualitative analysis. The time commitment for prompt development was retroactively estimated using query history provided in Python and processing times.

### Ethical Considerations

The data collection and analysis were deemed not human subjects by the University of Cincinnati Institutional Review Board (1/19/2023, MOD01_2021‐1032). Students consented for their responses to be used for platform development and general research purposes. The focus group transcript contained deidentified student responses and was stored in a secure, password-protected document.

## Results

### AI Inductive Coding Results

GPT-4o generated 137 codes through excerpt-by-excerpt analysis. Of the 137 AI codes, 31.4% (n=43) demonstrated “agreement” with human codes, 26.3% (n=36) were “reasonable alternatives,” and 42.3% (n=58) were “not reasonable,” including codes that were not textually supported or illogical.

Equally important were the codes the AI failed to identify: 21 out of 49 (42.9%) human codes were absent. Of note, the AI did not identify most human codes related to the clinical skills practiced or codes contrasting 2-Sigma with the real clinical environment. In contrast, it successfully identified codes focusing on desired features within 2-Sigma additions to increase the intuitiveness of 2-Sigma and additional skills that could be practiced in 2-Sigma.

### AI Deductive Coding Results

#### Calculating Interrater Reliability of AI vs Human Deductive Coding

GPT-4o assessed the transcript for the presence of the human codes across the transcript, yielding 2352 coding decisions (48 excerpts x 49 codes). Agreement between AI and human coders was quantified using percent agreement and Cohen κ. Across all codes, the mean percent agreement was 96% (SD 4%, range 79%‐100%) and the mean κ was 0.71 (SD 0.26, range 0‐1.0).

Cohen κ results are shown in Table 1. Out of 49 codes, 67.3% (n=33) of codes met the predetermined benchmark of κ>0.60, corresponding to “substantial” or “almost perfect” strength of agreement. All but 1 code met the predetermined benchmark of greater than 80% for percent agreement. Interrater reliability results are provided for all codes in Multimedia Appendix 5.

**Table 1.** Cohen κ results for the AI’s deductive application of the human codebook (N=49).

Number of codes, n (%)	κ range	Strength of agreement
0 (0)	<0.00	Poor
1 (2.0)	0.00‐0.20	Slight
7 (14.3)	0.21‐0.40	Fair
8 (16.3)	0.41‐0.60	Moderate
14 (28.6)	0.61‐0.80	Substantial
19 (38.8)	0.81‐1.00	Almost perfect

#### Misses and Misfires in AI Deductive Coding

Relative to human coding, out of 2352 coding decisions, GPT-4o correctly identified when a code was absent 2173 (92.4% of coding decisions) times. It correctly identified a present code 94 (3.9% of coding decisions) times. Error patterns included 57 (2.4%, inappropriate code applied) misfires and 28 (1.2% appropriate code not applied) misses.

#### Textual Analysis of Failures in AI Deductive Coding

To better understand GPT-4o’s specific limitations in deductive coding, we compared the source text, code, and reasoning for a subset of codes that tended to miss and a subset of codes that tended to misfire.

Codes most frequently missed by GPT-4o included those reflecting clinical skills (eg, history taking), contextual judgment (eg, valuable), and encounter dynamics (eg, prematurely ended encounter, lacks intraprofessional and interprofessional collaboration, and case responsiveness to student treatment decisions). Close review of GPT-4o coding decisions indicated 2 categories of recurring error patterns: failure to recognize key words and difficulty linking statements to their preceding context.

In several text excerpts, an obvious key word related to the code was present, but GPT-4o still failed to identify the code. For example, GPT-4o failed to code the excerpt containing “being able to practice that history-taking” for history taking. In another example, the student talked about increasing the realism of 2-Sigma by including collaboration with radiologists and pathologists, but GPT-4o failed to code the excerpt for lacks intraprofessional and interprofessional collaboration.

There were also a few instances where the text excerpt contained a continuation of an idea, requiring interpretation of the preceding text. In these cases, GPT-4o correctly coded the preceding, stronger example but failed to recognize the continuation of the idea. For example, 1 student described having her encounter cut short when she tried to explain her differential diagnosis to the patient. In the next text excerpt, she continued her idea, stating “I was just explaining that he was having a heart attack, right? [laughs]. So I think I was just trying to keep things going here.” For this excerpt, the code prematurely ended encounter was recognized by humans but not by GPT-4o.

The codes that tended to misfire (erroneous application) included abstract constructs (eg, synthesizing information), affective states (eg, excitement and confusion), and contextual judgment (eg, diagnosis is unclear and does not simulate real patient-physician communication). These examples illustrate that GPT-4o was prone to overinterpreting tone and treating hypothetical suggestions as actual experiences.

The code synthesizing information captures the clinical skill of piecing together various data points in 2-Sigma, such as the patient history, physical examination, and laboratory testing. However, GPT-4o often applied the code to excerpts that referred to hypothetical or suggested features rather than to actual student experiences in 2-Sigma. For example, GPT-4o applied the code synthesizing information to an excerpt where a student suggested check boxes to customize elements of the encounter, such as the history of present illness, past medical history, and assessment and plan. In doing so, GPT-4o missed the context of what was being said.

For the codes excitement and confusion, GPT-4o applied the codes more interpretively than humans. For example, GPT-4o applied the code “excitement,” defined as “students expressing excitement about possible additional features or use cases for 2-Sigma” to several lengthy excerpts where students described features that they would like to see. Although the humans did not initially recognize the code in these excerpts, on second review, many of them did contain an excited tone where the student response showed eagerness to describe all the possibilities. There were several times that GPT-4o contradicted itself in its coding and reasoning, applying a code but supplying a reason why the code did not apply, and vice versa.

### Implementation Resource Analysis

The manual thematic analysis required 34 coding hours across 3 human coders. In comparison, developing the AI workflow, including prompt design and infrastructure, took 65 hours, with a final AI run time of 80 minutes (30 min inductive; 50 min deductive). This 65 hours reflects the one-time cost of building and testing the workflow rather than a recurring cost.

## Discussion

### Principal Findings

#### Conceptual Framework for Evaluating AI Performance

Evaluating AI performance in qualitative coding presents a unique epistemological tension. While we use terms like “error” or “failure,” we acknowledge that human coding is not an objective ground truth but rather an interpretive, consensus-driven process. This study should be understood as a methods paper not a full qualitative study: we focused narrowly on the coding step to examine how AI performs relative to humans. We approached this by treating GPT-4o as a collaborator, engaging with its outputs critically to understand its strengths and limitations.

In the inductive condition, GPT-4o’s coding output often paralleled human interpretations, but it also produced tangential, textually unsupported, and illogical outputs that required careful human review. These observations are consistent with prior studies showing that LLMs can identify concrete patterns, but struggle with interpretive nuance [3,4,14], and therefore would benefit from collaboration with expert human adjudication to ensure validity. The AI can serve as an additional team member, proposing alternative codes and ensuring comprehensive coverage of the transcript, though it may miss constructs and context obvious to a human researcher.

In the deductive condition, the model demonstrated substantial agreement with human coders for most codes, comparable to what has been reported in other studies [1,2,15]. Its ability to achieve interrater agreement benchmarks reinforces its potential for scaling codebook-driven analyses [1]. However, its lower reliability for nuanced affective or context-dependent codes echoes concerns in the literature regarding AI’s difficulty with subtle, interpretive constructs [3,4,14]. This suggests that AI can efficiently handle labor-intensive, rule-based coding tasks but still requires human oversight for contextually complex codes. We found Cohen κ to be a more meaningful metric than percent agreement for evaluating AI performance, as high percent agreement was inflated by the large number of correctly identified negative instances (ie, code absence).

#### A Roadmap for AI Collaboration and Return on Investment

Our findings lead to a practical question: when is AI “good enough” for qualitative research? Mollick [16], in his work on AI in the workplace, suggests a useful heuristic: AI is worth using when its performance exceeds that of the “best available human” for a specific, bounded task. In our study, GPT-4o was accurate and efficient in laborious tasks like confirming the absence of codes within a large transcript and thus may be preferable to humans, who are prone to fatigue and error. However, for the nuanced task of identifying subtleties or distinguishing hypothetical suggestions from actual experiences, the human researcher currently has the edge.

This distinction suggests a roadmap: AI should not be seen as an autonomous replacement for the researcher but as a “cointellect” that can augment, but not replace, human analysis. Specifically, AI can provide coverage and efficiency at scale, while humans contribute interpretive depth and oversight.

**Figure 3.**
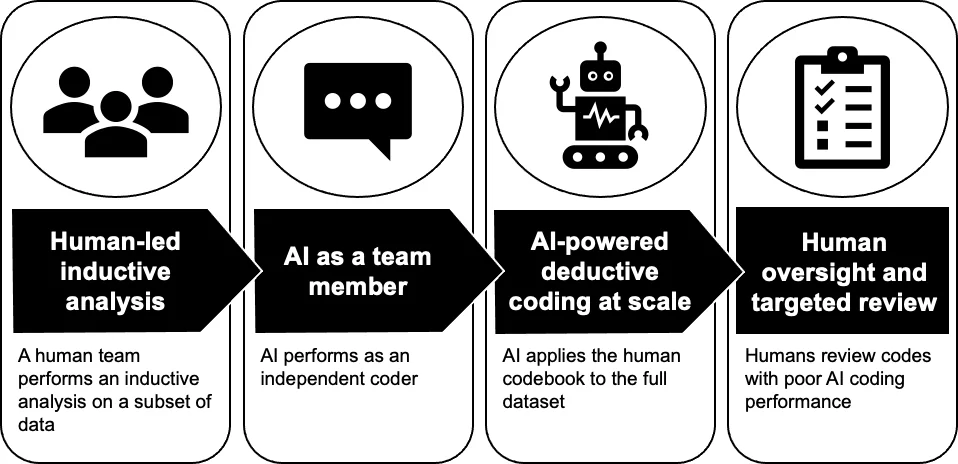
Human-in-the-loop workflow for qualitative analysis and coding. This figure demonstrates the components of our framework for effective human and AI collaboration: human-led inductive analysis, AI as a team member, AI-powered deductive coding at scale, and human oversight and targeted review. Our workflow incorporates human oversight, validation, and interpretive richness while leveraging AI for increased efficiency and scale.

To researchers interested in collaborating with AI for qualitative analysis, we suggest the following workflow, informed by our findings and Mollick principles (Figure 3):

1. Human-led inductive analysis: A human team performs the initial inductive analysis on a subset of data to develop a robust, contextually grounded codebook. This leverages the irreplaceable strength of human interpretation.
2. AI as a team member*:* The AI is then used to generate its own inductive codes on the full dataset. These are not taken as ground truth but as suggestions from a new team member, helping to identify alternative interpretations or codes that the human team may have missed. After human review of AI-suggested codes, a final codebook is created, representing both human and AI ideas, with an emphasis on human interpretive strengths. This approach balances the humans’ skills in inductive coding, with the acknowledgment of AI as a team member that may offer unique insight that humans missed due to coding fatigue or personal experiences biasing interpretation.
3. AI-powered deductive coding at scale: The human-validated codebook is provided to the AI to perform deductive coding of the full dataset. In this step, the strengths of AI in its deductive coding capabilities and opportunities for scalability are leveraged.
4. Human oversight and targeted review: Humans do not need to recode everything. Instead, they focus their efforts on reviewing the codes the AI was identified as being poor at handling (ie, those with low Cohen κ) and spot-checking a random sample of the AI’s work, a process Mollick [16] refers to as being the essential “human in the loop”.

This workflow optimizes collaboration by using AI for what it does best, scalable pattern matching, while reserving human expertise for nuanced interpretation and validation. Various frameworks for inductive qualitative coding exist, such as the thematic analysis framework of Braun and Clarke [10]. Our proposed workflow aligns with this method by suggesting the addition of an “additional researcher” to work alongside humans during the first 2 steps of the framework: familiarization with the data and code generation. In doing so, the AI may suggest codes that researchers overlooked or suggest alternative codes that may encourage further discussion among the human researchers.

Another method is generative AI-augmented thematic analysis (GAATA), which uses detailed prompting of AI for inductive code generation with subsequent human review. The AI then uses its human-validated codes to generate themes that are finalized by researchers [17]. Our method similarly leverages the efficiency of AI for inductive coding. However, in our method, humans code in parallel with the AI, with both generating initial codes from the source data. In GAATA, the role of the human in the inductive coding process is the review and refinement of AI-generated codes. In this regard, GAATA leverages the efficiency of AI inductive coding to decrease the time humans would spend inductively coding. Instead, we use AI’s efficiency not particularly to save human-hours but to serve as an “additional researcher” offering suggestions and perspectives that may have been overlooked in human coding and thus deepen the analysis. Additionally, our method does not address theme creation by AI. We focused on determining how to maximize AI’s abilities in the coding process, an essential step for later theme generation.

Another existing framework is conversational analysis to the power of AI, in which meaning is coconstructed through structured, iterative dialogue with LLMs rather than relying on more traditional systematic and segmental approaches to qualitative analysis [18]. Our workflow is similar in the aspect of a willingness to integrate AI interpretations of data and use these to guide discussions and offer a new perspective to think critically about our own coding decisions. Both also incorporate AI into the inductive and deductive steps of qualitative analysis. However, our workflow differs in the sense that it remains grounded in established coding-based workflows.

### Limitations

#### AI Prompt Engineering: Challenges and Mitigation Strategies

Efficiency gains via AI involvement in qualitative analysis were less than anticipated. Developing multistep prompts and automated infrastructure required nearly twice the time of a traditional 3-coder inductive analysis, contrasting with prior reports of efficiency advantages [3]. However, these setup costs are largely front-loaded; reusable prompts and infrastructure could substantially reduce effort in future applications. Implementation required advanced programming to automate logging, error handling, and formatting. This technical barrier may limit replication. To mitigate this, we documented our process and shared code to support adoption by other teams. Table 2 demonstrates the challenges encountered with AI prompting and the steps taken to mitigate them. The table’s purpose is to serve as an organized account of these challenges rather than describing conclusions regarding AI’s performance.

**Table 2.** Iterative refinement challenges and solutions.

Challenge	Solution	Result
Poor adherence to prompting instructions	Concise prompting without excessive detail; chain-of-thought, few-shot, and zero-shot prompting	Improved adherence to prompt
Skipped analysis of excerpts or codes	Splitting deductive coding task into individual code-excerpt pairs	Attention to all parts of transcript and all codes
Ignores steps in multistep prompts	Split steps into separate prompts	Improved adherence to each step
Poor contextual understanding	Included surrounding excerpts for additional context	Contextual limitations still evident in AI coding decisions
Variable emotional tone	Prompt engineering to encourage interpretation of emotions and sentiments	Increased emotional sensitivity
Incomplete outputs	Flexible, multistep fallback system that parses an AI-generated JSON output (via prompt) and recovers from output gaps by reprompting the AI for missing components	Logged full output of code, definition, and reasoning for every excerpt
Combined unrelated codes	Prompt engineering to favor a larger number of granular codes over a smaller number of imprecise codes	Codes maintained interpretive diversity; however, also greater redundancy
Inductive analysis initially combined codes based on proximity in transcript	Divorced codes from transcript, turned into list, and randomized order prior to inserting into prompt	Code groupings were more logical
Did not address all initial codes in final codebook	Assigned each initial code a number for automated tracking and automated a loop to identify missing codes iteratively	All initial codes were addressed in final codebook
Addressed the same initial code multiple times in codebook	Made output format extremely restrictive and processed the output to merge with, rather than overwrite, the existing codebook; decreased temperature of API call to decrease variability or “imagination” of AI	Each code was only addressed once in final codebook

Many of the challenges encountered were related to known limitations of LLMs. One such limitation is the difficulty of understanding without contextual information [19]. To mitigate this, we introduced a familiarization step, prompting the AI to summarize the full dataset before coding.

Contextual drift describes how as a task progresses, the AI can lose track of previous inputs or rules, leading to mistakes and inaccuracies. To assure the AI’s adherence to multistep instructions and maintaining attention across long texts [20-22], we divided complex processes into smaller tasks, and the analysis was performed using API calls from Python to GPT-4o so that no memory was retained between prompts.

Another limitation is hallucinations, in which outputs are stated confidently and as if grounded in data but are fabricated. To decrease hallucinations, the AI was required to provide a definition and a brief rationale with an example quote for each code. However, there were still instances in which the AI produced definitions and rationales not supported by textual evidence.

Last, AI sycophancy remains a significant limitation, as outputs are favored to match user preferences over truthfulness [23-26]. Although rationales were sometimes convincing and eloquent, they were not representative of the excerpt, consistent with broader concerns in AI research that models may generate convincing but unreliable reasoning [27,28].

One challenge encountered while creating the AI inductive coding process represented multiple limitations of AI. The 3-step process used in this study for AI-inductive coding initially incorporated an additional set of steps, which consisted of grouping similar codes, updating code definitions, and inserting a representative quote from the transcript. However, during that process, codes were separated from their source text, the source text was rephrased by the AI, and/or codes were paired with new nonsensical examples. This made the AI codebook difficult to evaluate and compare to that of the humans. Therefore, we decided to focus on the initially assigned codes which, although larger in number, maintained their connection to the original transcript. This preserved transparency in AI coding decisions, addressing the often opaque decision-making process of probabilistic models [2,29].

#### Study Limitations

This study has several limitations. First, we analyzed a single focus group transcript, which limited the breadth of data. Our familiarity with this transcript allowed a detailed comparison of AI and human coding, but broader datasets would provide a more comprehensive evaluation of AI performance. However, although the data may be limited in size, it represents a variety of characteristics seen in qualitative data analyzed in medical education. The situational context of statements shifted rapidly between experiences in the classroom, patient encounters, and independent study, all important parts of the medical student experience. Students also used key terms such as history-taking, differential diagnosis, and clinical skills. The transcript discussed not only logistical aspects of the student experience with 2-Sigma, such as the user interface or connectivity issues, but also addressed student emotions, both positive and negative, tied to learning.

A related limitation is that the human codebook was developed inductively from the same transcript to which it was later deductively applied by both human coders and AI. This raises a theoretical concern that deductive performance metrics could be inflated by direct overlap between codebook language and transcript content, rather than reflecting AI’s ability to generalize the codebook to unfamiliar data. However, this effect does not appear to fully explain our results. Our textual error analysis identified instances where GPT-4o failed to apply a code even when the excerpt contained language closely matching the codebook definition. Future work should evaluate deductive coding performance using codebooks and transcripts drawn from independent samples to more directly assess this effect.

Our study included only one model, GPT-4o, potentially limiting generalizability to newer models. As models are rapidly improving and evolving over time, it becomes challenging to evaluate and replicate findings during the window from model introduction to obsolescence.

Additionally, using participants’ speaking turns as the unit of analysis is coarser than coding at the level of discrete meaning units. Longer excerpts may contain multiple concepts and therefore provide more opportunities for codes to be applied. This unit choice can influence marginal code prevalence and estimates of interrater agreement. Interrater agreement should be interpreted as agreement on whether a code was present within a participant turn, rather than meaning-unit-level agreement.

Last, our comparison of coding time between humans and AI reflects only the one-time cost of developing the AI workflow and applying it to a single transcript. Future work should evaluate the time required to apply an already-validated AI workflow to new transcripts, which may better capture its efficiency at scale.

### Conclusions

The emergence of AI has prompted researchers to investigate how it could best be used in qualitative analysis. Current literature suggests various strategies that differ in their level of reliance on AI in the coding process and some strategies that use AI to reimagine the qualitative coding process altogether [17,18]. However, few studies have evaluated AI’s performance in these areas within the context of medical education, where maintaining interpretive rigor is especially important, as qualitative data containing unique concepts, rich insight, and affective range are used to guide curricular decisions. Fewer have evaluated AI’s performance in this specific context through comparison with human analysis [7,8].

Our study directly compared human and AI performance in deductive and inductive coding of qualitative data to lead us to a proposed workflow for AI in qualitative analysis that focuses on the human perspective and decision-making. Our workflow emphasizes the role of AI as that of an additional perspective to aid in human-driven reflexive analysis rather than emphasizing AI’s capabilities for increased efficiency. We believe this approach best balances insight with scalability for researchers interested in integrating AI into medical education qualitative research.

## Supplementary material

10.2196/85572 Multimedia Appendix 1 Full human-generated codebook.

10.2196/85572 Multimedia Appendix 2 Full inductive prompts.

10.2196/85572 Multimedia Appendix 3 Example and rationale for each category of alignment of AI inductive codes.

10.2196/85572 Multimedia Appendix 4 Full deductive prompts.

10.2196/85572 Multimedia Appendix 5 Interrater reliability of the AI deductive application of human codebook. Cohen κ and percentage agreement are shown for each code.

## Abbreviations

GAATA: generative AI-augmented thematic analysis
LLM: large language model
