# Supplementary material for: Large Language Model–Assisted Thematic Coding in Medical Education Research: Comparative Methodological Study
Source: JMIR Form Res. 2026 Aug 25;10:e85572. doi: 10.2196/85572 (PMC13505884; doi:10.2196/85572)
Supplement: Supplementary file 1 [file formative-v10-e85572-s001.pdf]

Theme	Code	Description	Example 1
Clinical Skills Practiced	History Taking	Students describing or stating how they used 2-Sigma for history taking skills, including asking the patient questions about their current problem (the HPI), review of systems, past medical history, or social history.	(ex). "based on their presentation and responses for any questions that you asked"
	Creating a Differential Diagnosis	Students describing or stating how they used 2-Sigma for generating a differential diagnosis, including conceiving of possible explanations for the patient's presentation, sorting possible explanations by likelihood, and eliminating items that are less likely.	(ex). "practicing ... this was higher than that, and then you eliminate something based on that result"
	Differential Guided Workup	Students describing or stating how they used 2-Sigma to practice adapting their history taking, physical exam maneuvers, or laboratory testing based on their differential diagnosis.	(ex). "Which systems should I think about? ... And then go on from there by tailoring the questions ... Then, appropriate lab testing afterwards once you've reached which system you should be testing in."

	Synthesizing Information	Students describing or stating how they used 2-Sigma to practice piecing together all data points from the encounter, such as the patient history, physical exam, and laboratory testing.	(ex). "trying to put pieces of information together"
	Patient-Centered Communication	Students describing or stating how they used 2-Sigma to practice explaining a possible diagnosis, thought process and/or next steps to the patient. This does not include communication for the purpose of gathering a history.	(ex). "In like, two or three cases, I was just trying to explain to the patient, 'Hey, I think this might be a possibility. This might be a possibility. I'm testing for this because of this.'"
	More Time to Think	Students describing the ability to take extra time working through the case while using 2-Sigma.	(ex). "because you have more time, you can choose to hit the button or not. I think it's a good way to organize your thoughts without it being like in clinical skills where they're right there and you have seconds to think."
	Repetition	Students describing the ability to learn through repetition while using 2-Sigma.	(ex). "it gives us a chance to see more examples"

Advantages Specific to 2-Sigma	Comprehensive	Students explicitly stating the ability to practice increased breadth or depth of the elements of the patient encounter in 2-Sigma, relative to other learning settings. This code is not implied by discussing a variety skills practiced in 2-Sigma.	(ex). "I did feel like it helped me gain confidence like doing more than just with my team and people in clinical skills."
	Multiple Systems	Students describing the ability to think through multiple organ systems when completing cases in 2-Sigma, in contrast to other learning settings which are organized by organ system block.	(ex). "A lot of the time when we're in a certain block, you have that mindset of you're working hard in this block, so this can be a cardio-related case. And I'm sure this, the Sigma app can go beyond that as it develops. But it really helped me go into a different mindset of just like a hierarchy of like, okay, this is the patient presenting. Which systems should I think about? Should it be cardio only? Should it be cardio and pulm? Should it be neuro?"
	Independence	Students describing the ability work through the case on their own in 2-Sigma.	(ex). "I liked how it gave more opportunity to do that by yourself, because in clinical skills we're doing it with three people."
Positive Student Reactions	Valuable	Students acknowledging the value of 2-Sigma in their education. This code does not apply to students discussing the potential value of suggested features.	(ex). "it's a nice tool in that sense"
	Excitement	Students expressing excitement about possible additional features or use cases for 2-Sigma.	(ex). "Especially if it was able to give more specific feedback ... That would be really cool. "

User Interface Weaknesses	No Enter Message Button	Students discussing the lack of an enter button for submitting messages while completing the case. This code is specific to the act of continuing the encounter. It is separate from the topic of ending the encounter.	(ex). "I just want to be able to hit enter and send the message."
	Prematurely Ended Encounter	Students discussing instances where the app ended the encounter too early.	(ex). "there were some times, like two or three cases, I was just trying to explain to the patient, hey, I think this might be a possibility…and it shut down the case. And I was like, I wasn't done"
	Poor Connectivity	Students discussing connectivity issues while using 2 Sigma.	(ex). "Also the connectivity issues"
	Unclear Who Is Talking	Students discussing poor demarcation between the patient speaking and the AI prompts.	(ex). "One thing that I thought was a little bit confusing platform was when it has a patient response, and then it said, 'what would you do next?' It wasn't like any kind of demarcations."
	Frustration	Students describing frustration while interacting with 2-Sigma.	(ex). "that was a little tedious"
	Confusion	Students describing confusion while interacting with 2-Sigma.	(ex). "One thing that I thought was a little bit confusing..."

Unenjoyable Learning Experience	Unnecessary Practice	Student stating or implying that elements of the 2-Sigma cases are unhelpful because the practiced skills are covered elsewhere in the curriculum, or not transferable to real clinical environment.	(ex). "because that is kind of the part that we focus on the most in LC. Also clinical skills, we do a lot of that very initial part. ... everyone can do severity one out of 10 by now."
Feedback Limitations	Diagnosis is Unclear	Students discussing desire for a definitive correct diagnosis in the feedback for the 2-Sigma case.	(ex). "I am a little confused about what the actual diagnosis is."
	Diagnosis is Too General	Students discussing desire for more detailed diagnosis for the 2-Sigma case.	(ex). "they'd be like 'Yeah it was pneumonia'. Then that's super vague. Was it viral, was it bacterial?"
	Feedback Not Contextual	Students discussing instances where the feedback in 2-Sigma was not appropriate to the circumstances of the case.	(ex). "One thing that stuck out to me was like some scenarios who have something urgent going on, like they're in the middle of a heart attack. And then the feedback would be, 'You should have spent more time discussing their lifestyle.' I don't think that's the best time to talk to them about that."
	Lacks Intraprofessional and Interprofessional Collaboration	Students discussing the role of other health care professionals in the real clinical environment, including supervising physicians, physicians in other specialties, and colleagues such as nurses.	(ex) "A lot of times in FOD, it's very rare that I'm going into a patient room uninformed, because the nurses come up to me and say, 'Oh, hey, they have a sore throat' or 'They have an infected toenail.' The nurse generally will come up to us and give us some type of input."

Dissimilar From Real Clinical Environment	Lacks Collateral History	Students discussing other sources of patient history in the real clinical environment, including other professionals, family, or the chart.	(ex). "I'm more likely to ask whoever brought the kid or person to the hospital for collateral history. Because the person can't breathe, I'm not going to ask them for information right off."
	Lack Visual Clues to Diagnosis	Students contrasting the lack of visual input in 2-Sigma to the real clinical environment.	(ex). "Having an actual person there is cueing a lot of different things other than just reading all the texts."
	Does Not Simulate Real Patient Physician Communication	Students contrasting interacting with the platform to communicating with real patients.	(ex). "I wasn't going to say to the patient 'order CBC.'"
	Multimedia	Students discussing the addition of multimedia such as images or sound to 2-Sigma.	(ex). "You could do echocardiograms. Maybe starting with the imaging part of it or little snapshots of stuff."
	Preceptor	Students discussing desire for a preceptor within the 2-Sigma case to provide further prompting/questioning in response to student assessment and decisions.	(ex). "then the AI prompts and says, 'Okay, well, why?' maybe just explaining yourself, and then saying, 'Okay, what would you do next?' ... this might dig even deeper into it, like, 'Why do you think it's a heart attack?' And then 'What other things are you concerned about? What are other things that you don't want to miss on this differential?' Kind of like, prompting you."

Desired Features Within 2-Sigma	Student Sets Objectives	Students discussing features in 2-Sigma that would allow the student to tailor the objectives and scope of the case, including specifying their own role in the case or entering the case and ending the case at different points.	(ex). "depending on what your goals for the assignment are, if you are done, press the 'I'm done' button. But if, if you are curious about more of it ... it could prompt you a little more"
	Provide HPI	Students dicussing the option to have entire or partial HPI provided at beginning of the 2-Sigma case.	(ex). "If you have a practice session box and underneath that's like, provide us with OLDCARTS, HPI, whatnot."
	Case Responsiveness to Student Treatment Decisions	Students discussing potential for 2-Sigma to reflect patient progression following student treatment decisions.	(ex). "instead of like, ending the case, it's like, 'Oh, they come back two weeks later and they're worse,' instead of better. And then what do you do?"
	After-Case Information	Students discussing teaching points about the condition and/or its treatment to be provided after the 2-Sigma case is completed.	(ex). "Then maybe have something that says a full story to conclude that it was a case of viral pneumonia. They got it because of this, you should've expected this when you saw this."
	Clear End Point	Students discussing alternative ways to end the case in 2-Sigma.	(ex). "having a phrase to mark the end of the case"
	Pause Button	Students discussing the ability to pause during a case in 2-Sigma and resume at a later time.	(ex). "But I know some people when they were doing the actual case itself, like if you got a phone call or something ... And you came back. It just made you redo the entire thing."

Increase Intuitiveness	Out-Of-Chat Actions	Students discussing actions that could be performed outside of the chat format, such as lab ordering.	(ex). "because everything was in chat mode, I think it was a little bit weird….If there was a separate box that said, you know, I want labs and I want to order whatever lab are you, you type it in there."
	Define AI Roles	Students discussing ways to demarcate between the different roles of the AI in 2-Sigma: voicing the patient, moderating, and precepting.	(ex). "If we implement something [where] there's a patient character and there's a preceptor or a moderator character."
Additional Skills to Include in 2-Sigma	More Sophisticated Differentials	Students discussing the incorporation of more detailed differentials in 2-Sigma, such as prioritizing and justifying the differential, considering most common causes and "can't miss" (severe, reversible) diagnoses.	(ex). "and then the AI prompt goes, 'what are other things that you don't want to miss on this differential?'"
	SOAP Notes	Students discussing the incorporation of SOAP notes into 2-Sigma.	(ex). "SOAP notes I think…would be a big thing."
	Oral Presentations	Students discussing delivering oral presentations in 2-Sigma.	(ex). "Presenting would be a big thing."
	Difficult Patient Conversations	Students discussing delivering bad news or answering difficult patient questions in 2-Sigma.	(ex). "also delivering diagnoses that are like, 'Oh you're having a heart attack,' and then it ends or whatever. But then it's like how do you tell the patient they're having a heart attack? And then have to be able to ask like questions like, 'Oh am I gonna die?'"

	Medical Management	Students discussing the incorporation of medical management into 2-Sigma, such as stepwise diagnostics and treatment.	(ex). "where we don't get as much practice and where I would some value [is] going in deeper on the assessment and plan"
Use Cases for 2-Sigma Clinical Scenario Tool	OSCE Preparation	Students discussing using 2-Sigma cases to prepare for OSCEs (Observed Structured Clinical Examinations).	(ex). "Like a practice test for an OSCE. ... You could say an area or some symptoms that you think you might have [on the OSCE]. And then, they generate a story and the test results and stuff. I could definitely see that being practice for what to do. Especially if it was able to give more specific feedback."
	Clinical Coursework	Students discussing using 2-Sigma cases to augment clinical curriculum in M3 and M4 years.	(ex). "I don't know if you guys have also integrated with clerkships, but I know I've been talking to M3s recently and there's a lot of busy work assignments that are just not helpful .... these could be assignments that replaced those assignments where you're actually learning things or practicing skills that could be helpful for day to day work."
Desired AI Learning Tools Outside of Clinical Scenarios	Lecture Aid	Students discussing a feature in 2-Sigma that would create a short synopsis of lecture videos and generate test questions.	(ex). "Or take the question based on the lecture. It's not necessarily the questions are building for testing, but ... more like making sure you actually absorb that information."
	Personal Learning Companion	Students discussing the addition of a personal learning companion to 2-Sigma, to whom students could ask questions related to lecture content.	"I mean, I use ChatGPT to ask questions about lectures......it'll give me, you know, a better answer than Google..... so I mean, I can take that from ChatGPT to this personal learning companion pretty easily."

	Spaced Repetition	Students discussing the addition of recall questions with automated spaced repetition to 2-Sigma.	(ex). "And then if you like, get the question wrong, then it could come back up, kind of like an Anki situation."
Challenges of Adopting New Learning Tool	Time Constraints	Students discussing difficulty completing cases in 2-Sigma due to time constraints.	(ex). "It's hard for me to be like, I'm gonna you know, do this extra thing. And that doesn't really count."
	Ungraded	Students stating or implying that students would be unlikely to complete clinical scenarios without an associated grade.	(ex). "I couldn't necessarily see myself doing clinical scenarios because there's no clinical skills grade on it. ... I'd love to do it to better myself, and we just don't have the time to do that."
	Not Immediately Relevant	Students stating or implying that 2-Sigma focuses on skills that are not immediately relevant to first- and second-year medical students.	(ex). "in M1, M2, we're not in the wards yet, where it's actually immediately relevant and applicable."
