# Supplementary material for: Large Language Model–Assisted Thematic Coding in Medical Education Research: Comparative Methodological Study
Source: JMIR Form Res. 2026 Aug 25;10:e85572. doi: 10.2196/85572 (PMC13505884; doi:10.2196/85572)
Supplement: Supplementary file 2 [file formative-v10-e85572-s002.pdf]

Full Inductive Prompts

GitHub: https://github.com/zahn3001/Jennings_Zahn_QualitativeAnalysis/tree/main

Step 1: Familiarization with Transcript

```
# Define the full Braun & Clarke excerpt as a separate object
braun_clarke_excerpt = """
Phase 1: Familiarization involves immersing yourself in the data through repeated reading and active engagement to identify initial patterns, meanings, and ideas.
Avoid skipping content; this step lays the foundation for coding. Begin taking notes but avoid formal coding at this stage.
"""

# System Message defining AI's role and methodology
system_message = {
"role": "system",
"content": (
"You are an AI assistant trained to perform thematic analysis based on Braun & Clarke's 6-phase framework.\n\n"
"**Current Focus: Step 1 – Familiarization with the Data**\n"
"- Read the entire dataset thoroughly (repeated reading encouraged).\n"
"- Look for recurring ideas, broad topics, and initial paterns.\n"
"- Do not perform coding or theme categorization yet.\n"
"- Take note of key concepts or areas of interest for future analysis.\n"
"- This summary will serve as a foundation for more detailed analysis later."
)
}


# Process Step 1 (Familiarization with Data)
def process_step():
user_message = {
"role": "user",
"content": (
"Step 1: Familiarization with Data\n\n"
"Review the dataset and provide a high-level summary of broad themes, patterns, and key ideas. Follow Braun & Clarke's methodology for familiarization (2006) as described below:\n\n"
f"{braun_clarke_excerpt}\n\n"
f"**Dataset:**\n{file_content}\n\n"
"### **Expected Output:**\n"
"- A structured summary of key themes, patterns, and conversation topics.\n"
"- Ensure the output aligns with the familiarization phase (repeated reading, note-taking).\n"
"- Avoid specific codes or formal theme names at this stage."
)
}

response = client.chat.completions.create(
model="gpt-4o",
messages=[system_message, user_message],
response_format={"type": "text"},
temperature=1,
max_tokens=8192
```

```
)
```

Step 2: Generating Initial Codes

```
# System Message defining AI's role and methodology
system_message = {
"role": "system",
"content": (
"You are an AI assistant and expert in medical education, trained to conduct thematic analysis using Braun & Clarke’s 6-step methodology.\n\n"
"**Current Focus: Step 2 – Generating Initial Codes**\n"
"- Identify and label meaningful features in each student response.\n"
"- A 'code' is a brief phrase capturing a significant idea related to the research aim.\n"
"- Focus on content, contradictions, and nuances—do not reduce to themes.\n"
"- Treat each data segment with equal importance.\n"
"- Work iteratively and avoid assumptions or biases.\n"
"- Use clear, concise language to generate consistent, comparable codes."
)
}

# AI prompt
user_message = {
"role": "user",
"content": (
"Step 2: Generating Initial Codes\n\n"
"Generate 1–3 concise codes that capture the key ideas or sentiments in the following student response. "
"Think iteratively—consider meaning, nuance, emotions, sentiments, and contradictions before deciding on codes. "
"Refer to Braun & Clarke’s guidance for initial coding and use the familiarization summary for context.\n\n"
"### Step 1 Familiarization Summary:\n"
"Use the following summary to understand the broader context of the data and help you infer meaning from responses that may seem ambiguous without additional context:\n\n"
f"{step1_output}\n\n"
"### Student Response:\n"
f"\"{student_text}\"\n\n"
"### Output Instructions:\n"
"- Respond using strict JSON format only.\n"
"- Return a list of dictionaries (maximum of 3).\n"
"- Each dictionary must contain:\n"
" - \"code\": <short label>\n"
" - \"definition\": <brief description>\n"
" - \"reasoning\": <why it fits the student response>\n\n"
"### Example:\n"
"[\n"
" {\n"
" \"code\": \"Feedback Loop Awareness\",\n"
" \"definition\": \"Recognizing how feedback from the AI influences learning\",\n"
" \"reasoning\": \"The student describes how immediate feedback changed their understanding.\"\n"
" },\n"
" {\n"
" \"code\": \"Confidence Building\",\n"
" \"definition\": \"Gaining assurance through AI-based practice\",\n"
" \"reasoning\": \"The student mentions that using the tool helped them feel more prepared.\"\n"
" }\n"
"]"
)
}
```

```
# Send request to OpenAI
response_ai = client.chat.completions.create(
model="gpt-4o",
messages=[user_message],
response_format={"type": "text"},
temperature=1.25,
max_tokens=750
)
```

Step 3: Final Codebook Generation

```
# ===== Prompt #1: Final Code Grouping and Definition Refinement =====
system_message = {
"role": "system",
"content": (
"You are an expert qualitative researcher and expert in medical education trained to conduct rigorous qualitative analysis following Braun & Clarke's (2006) thematic analysis guidance tasked with creating **final codes** from a set of initial codes and their definitions."
"You have completed the first two steps of thematic analysis: familiarization with the data and generating initial codes."
"### High-Level Context from Step 1 Familiarization (summarization of transcript)\n"
f"{step1_context}\n\n"
"A **final code** is a short, precise label that captures a specific, observable idea or concept in the data. It is concrete, descriptive, and based on what is explicitly stated in the responses."
"Final codes are **NOT** themes. A theme is a broader, interpretive concept that groups multiple codes together to tell a larger story; final codes remain more specific and granular."
"Your role is to:"
"- **Group** related initial codes into the most fitting final code(s)"
"- **Create** a new final code only if the initial code does not fit any existing final code"
"- Ensure each final code has a clear, concise **definition** that captures its meaning without expanding into theem-level interpretation."
"- Follow all requested output formats exactly."
"Always avoid producing themes or broad categories - focus only on generating the most accurate set of **final codes** and their definitions."
)
}


user_message = {
"role": "user",
"content": (
"""You are reviewing 50 initial codes and definitions from a qualitative analysis of a focus group transcript. You have already seen a high-level summary of the transcript.


Your task is to synthesize the **most obvious** groupings into final codes. A final code is specific, concrete, and descriptive (not a broad theme). Only group codes that clearly share the same idea. If a code does not clearly fit, leave it ungrouped for later refinement.Take into consideration implicit meaning in both initial and final code names and definitions when making your decision.


**Instructions**
- Review **all 50** initial codes before grouping; weigh them equally.
- Create final codes only for **clear, high-confidence groupings** based on explicit AND implicit shared meaning between initial code names or definitions.
- You may leave any number of initial codes ungrouped at this stage.
- Do **not** invent or assume content beyond the provided code names/definitions.
- Use your own concise wording for each final code's name and definition.
- Each initial code number may appear in **at most one** final code (no duplicates across groups).
- **Do not include** any initial code numbers that are not in the provided list.
- Keep the focus on specific, observable ideas (final codes), **not** broader interpretive themes.


**Output Format (EXACTLY)**
For each final code you decide to produce, output a block in this exact format:
```

```
**Code: [Final Code Name]**
**Definition:** [Concise definition grounded only in the data]
Grouped Codes: 1, 4, 7
Explanation: [1–2 sentences explaining the shared idea and why these codes belong together]

Formatting rules:
- `Grouped Codes:` must be plain comma-separated integers with **no brackets or extra text** (e.g., `1, 4, 7`).
- Do **not** include any other headers, lists, or commentary outside the blocks.
- It is acceptable to output only a few blocks if only a few groupings are clearly obvious.

**Quality checks (apply before returning your answer)**
- No bracketed numbers (e.g., `[1]`) and no duplicates across any groups.
- Every number under `Grouped Codes:` must be from the provided list and may appear once at most.
- Each block has all four lines in the exact order and wording shown above.
IMPORTANT: Output grouped code numbers as plain comma-separated integers with no brackets or extra text (e.g.,
`Grouped Codes: 1, 4, 7`).\n\n"
### Initial Codes:\n\n"""
"```" + full_code_text + "```"
)
}


response_ai = client.chat.completions.create(
model="gpt-4o",
messages=[system_message, user_message],
response_format={"type": "text"},
temperature=0.5,
max_tokens=9000
)

# === Prompt 2: Codebook Refinement for Unused Codes ===
system_message = {
"role": "system",
"content": (
"You are an expert qualitative researcher (medical education) applying Braun & Clarke’s (2006) approach. "
"You have completed familiarization and initial coding. The goal now is to refine **final codes**. Take into consideration
implicit meaning in both initial and final code names and definitions when making your decisions.\n\n"
"DEFINITIONS\n"
"- A **final code** is a short, specific, concrete label that captures an observable idea explicitly present in the data. "
"It is not interpretive or thematic; it is precise and descriptive.\n"
"- A **theme** is broader and interpretive and is NOT what you should produce.\n\n"
"DECISION RULES (binary)\n"
"1) If the initial code clearly fits an existing final code’s definition, **assign it to that final code**.\n"
"2) Create a **new final code** only if the concept is **clearly not** captured by ANY existing final code definition.
Consider ALL existing final codes in this step.\n"
"If uncertain after comparison, **assign to the closest existing final code** (conservative default).\n\n"
"CONSTRAINTS\n"
"- Do NOT modify or remove any existing final code names, definitions, or grouped codes.\n"
"- You may only (a) append the current initial code number to one existing final code, or (b) add one new final code.\n"
"- Output must match EXACTLY one of the two formats below and NOTHING ELSE.\n"
"- When you include grouped code numbers, use plain comma-separated integers with no brackets or extra text.\n\n"
"STEP 1 CONTEXT (high-level familiarization summary used to ground decisions)\n"
f"{step1_context}\n"
```

```
)
}

prompt = (
"You are reviewing ONE initial code and its definition, and the list of existing final codes.\n\n"
"Your task: Decide between exactly two options:\n"
"A) Assign this initial code to an existing final code (if it clearly fits), OR\n"
"B) Create ONE new final code (ONLY if it is clearly not covered by ANY existing final code definition).\n"
"If uncertain after comparing definitions, **assign** to the closest existing final code.\n\n"
"You must return EXACTLY ONE of the two formats below and NOTHING ELSE.\n\n"
"FORMAT 1 — Assign to existing (single line):\n"
"assigned to existing final code ##\n\n"
"FORMAT 2 — Propose ONE new final code (exactly 4 lines):\n"
"**Code: [Final Code Name]** \n"
"**Definition:** [Concise, concrete definition] \n"
"Grouped Codes: [number] \n"
"Explanation: [Which existing final code was closest and why it did not apply]\n\n"
"EXAMPLES (good):\n"
"assigned to existing final code 12\n\n"
"**Code: Clarifying Follow-up Questions** \n"
"**Definition:** Student explicitly asks targeted follow-up questions to refine the differential. \n"
"Grouped Codes: 57 \n"
"Explanation: Closest was row 9 (History Taking), but that focuses on breadth of initial questions rather than targeted
follow-ups.\n\n"
"BAD EXAMPLE (do NOT do this):\n"
"“It might be row 12 or 14, and we could also create a new code called X…” ← Not allowed. You must choose one
option.\n\n"
"REMINDERS\n"
"- Do not alter existing final code names/definitions/grouped codes.\n"
"- Use only plain integers for grouped codes (no brackets).\n"
"- Return exactly one of the two formats; no extra commentary.\n\n"
"- Consider ALL existing final codes before deciding to create a new one.\n\n"
"#### Initial Code:\n"
f"{unused_codes_text}\n\n"
"#### Existing Final Codes (with row numbers):\n"
f"{current_grouped_codes_reference}\n"
)

response_ai = client.chat.completions.create(
model="gpt-4o",
messages=[system_message, user_message],
response_format={"type": "text"},
temperature=0.2,
max_tokens=1500

# Prompt 3: Quote Selection
system_message = {
"role": "system",
"content": (
"You are an AI assistant trained to conduct rigorous qualitative analysis. "
"Your task is to identify the most representative student quote from the transcript for a given final code and its definition.
"
"You must give EQUAL consideration to every student quote before making your selection."
)
```

```
}

user_message = {
"role": "user",
"content": (
f"### Final Code and Definition\n"
f"**Code:** {code_name}\n"
f"**Definition:** {definition}\n\n"
"You must select a short quote (1–2 sentences maximum) from the transcript that specifically and clearly reflects the final code and its definition. \n"
"Give EQUAL consideration to every student quote before making your selection.\n\n"
"### Instructions for Quote Selection:\n"
"Only include the relevant portion of the student response that directly pertains to the final code — do not return the entire student response.\n\n"
"Return only the selected excerpt — no commentary or headers.\n"
"At the end of the quote, include the transcript block number from which it was taken in the format: (ID: ##)\n\n"
f"### Transcript:\n{transcript_reference}"
)
}

response = client.chat.completions.create(
model="gpt-4o",
messages=[system_message, user_message],
response_format={"type": "text"},
temperature=0.5,
max_tokens=300
)
```
