# Supplementary material for: Large Language Model–Assisted Thematic Coding in Medical Education Research: Comparative Methodological Study
Source: JMIR Form Res. 2026 Aug 25;10:e85572. doi: 10.2196/85572 (PMC13505884; doi:10.2196/85572)
Supplement: Supplementary file 3 [file formative-v10-e85572-s003.pdf]

AI Codebook			Human Codebook		Alignment Category	Comment
Code	*Definition*	*Reasoning*	*Code*	*Definition*		
**Independent Practice**	Ability to practice clinical skills autonomously outside sessions	The student values 2-sigma for allowing them to practice independently beyond scheduled clinical sessions, highlighting self-directed learning.	**Independence**	Students describing the ability work through the case on their own in 2-sigma.	Agreement	This code matches the human generated code "independence" very closely
**Diagnostic Process Understanding**	The practice of synthesizing information to reach differential diagnoses	The student discusses the analytical approach to evaluating clinical data and determining which conditions are more likely than others.	**Creating a Differential Diagnosis**	Students describing or stating how they used 2-sigma for generating a differential diagnosis, including conceiving of possible explanations for the patient's presentation, sorting possible explanations by likelihood, and eliminating items that are less likely.	Agreement	This code essentially captures the process of building a differential, but there is also overlap with the idea of data synthesis, which we delineated into a separate code
**Concept Approval**	Positive sentiment towards the overall idea or concept of the tool.	The student's response begins with enthusiasm for the general concept, indicated by their expressions of liking it.			Reasonable Alternative	We captured "valuable", when student discusses features being helpful for their education, and "excitement" referring to excitement about suggested features, but not approval
**Interactive Learning Enhancement**	Enhancing learning through interaction and case customization	The response discusses the method of tailoring questions and approaches within the app, promoting an interactive and adjustable learning experience across various medical insights.			Not Reasonable	This code is difficult to understand. It seems to misinterpret "tailoring the questions", referring to student questioning the patient to refine their differential, as referring to tailoring 2-sigma itself
