# Supplementary material for: Large Language Model–Assisted Thematic Coding in Medical Education Research: Comparative Methodological Study
Source: JMIR Form Res. 2026 Aug 25;10:e85572. doi: 10.2196/85572 (PMC13505884; doi:10.2196/85572)
Supplement: Supplementary file 4 [file formative-v10-e85572-s004.pdf]

Full Deductive Prompts

GitHub: https://github.com/zahn3001/Jennings_Zahn_QualitativeAnalysis/tree/main

Deductive Coding Prompt:

```
prompt = f"""
You are a qualitative coding assistant. Given a single target student's response and some surrounding context, determine whether the following code applies to the student's response.

### CODE:
{code}
Definition: {desc}
Example: {ex}

{context_block}

### INSTRUCTIONS:
Consider whether the code meaningfully applies to the **target student's response**, using the definition and example as guidance. Read the entire **target student's response**, paying equal attention to each line, and understand the nuance and context of the quote. Use the **code definiton** to fully understand the intent and meaning of the code and use the **example** to give further context to your understanding of the code. You may apply the code when there is **clear relevance or strong implication** in the **student's response**, even if not stated explicitly. You should apply the code if the code is obviously present in the target student's response. Do **not** apply a code based on what other students say in the surrounding context. If the code applies, explain in 1–2 sentences why it fits. If it clearly does not apply, reply exactly: "0". Interpret the code with care, but do not be overly restrictive — aim for reasonable, justifiable matches to the target response.
"""

response = client.chat.completions.create(
model="gpt-4o",
messages=[{"role": "user", "content": prompt}],
temperature=0.4,
max_tokens=300
)
```
