# Supplementary material for: Large Language Model–Assisted Thematic Coding in Medical Education Research: Comparative Methodological Study
Source: JMIR Form Res. 2026 Aug 25;10:e85572. doi: 10.2196/85572 (PMC13505884; doi:10.2196/85572)
Supplement: Supplementary file 5 [file formative-v10-e85572-s005.pdf]

Code	Κ	% Agreement
History Taking	0.54†	94
Creating a Differential Diagnosis	0.73	96
Differential Guided Workup	1.00	100
Synthesizing Information	0.36†	88
Patient-Centered Communication	0.48†	96
More Time to Think	0.38†	94
Repetition	0.64	96
Comprehensive	0.45†	92
Multiple Systems	1.00	100
Independence	1.00	100
Valuable	0.54†	94
Excitement	0.34†	88
No Enter Message Button	1.00	100
Prematurely Ended Encounter	0.48†	96
Poor Connectivity	0.85	98
Unclear Who Is Talking	1.00	100
Frustration	0.78	96
Confusion	0.23†	79†
Unnecessary	0.29†	92
Diagnosis Is Unclear	0.48†	96
Diagnosis Is Too General	0.79	98
Feedback Not Contextual	1.00	100
Lacks Intraprofessional and Interprofessional Collaboration	0.00†	94
Lacks Collateral History	0.66	98
Lack Visual Clues to Diagnosis	0.66	98
Does Not Simulate Real Patient-Physician Communication	0.54†	94
Multimedia	1.00	100
Preceptor	0.45†	92
Student Sets Objectives	0.79	94
Provide HPI	1.00	100
Case Responsiveness to Student Treatment Decisions	0.37†	94
After-Case Information	0.64	96
Clear End Point	0.65	96
Pause Button	1.00	100
Out-Of-Chat Actions	0.66	98
Define AI Roles	0.65	96
More Sophisticated Differentials	0.79	98
SOAP Notes	1.00	100
Oral Presentations	1.00	100
Difficult Patient Conversations	1.00	100
Medical Management	0.85	96
OSCE Preparation	1.00	100
Clinical Coursework	1.00	100
Lecture Aid	0.79	98
Personal Learning Companion	0.48†	96
Spaced Repetition	1.00	100
Time Constraints	1.00	100
Ungraded	1.00	100
Not Immediately Relevant	0.66	98

† Denotes code not meeting benchmark of >0.60 for Cohen’s κ or >80% for percent agreement.
